# Supplementary material for: Development of an Organ-Directed Exosome-Based siRNA-Carrier Derived from Autologous Serum for Lung Metastases and Testing in the B16/BL6 Spontaneous Lung Metastasis Model
Source: Pharmaceutics. 2022 Apr 7;14(4):815. doi: 10.3390/pharmaceutics14040815 (PMC9025519; doi:10.3390/pharmaceutics14040815)
Supplement: Supplementary file 1 [file pharmaceutics-14-00815-s001.zip › pharmaceutics-1615594-supplementary.pdf]

Supplementary Information

Development of an organ-directed exosome-based siRNA-carrier derived from autologous serum for lung metastases and testing in the B16/BL6 spontaneous lung metastasis model

Mai Hazekawa[1*], Takuya Nishinakagawa[1], Masato Hosokawa[1] and Daisuke Ishibashi[1]

[1]Department of Immunological and Molecular Pharmacology, Faculty of Pharmaceutical Sciences, Fukuoka University, Fukuoka, Japan

Supplementary Figure S1

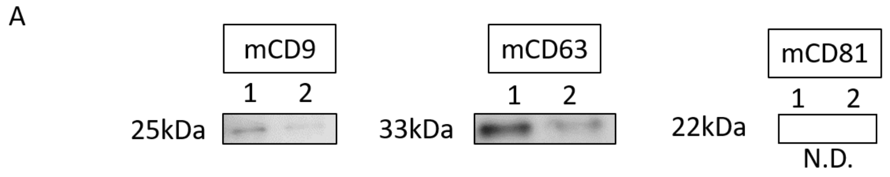

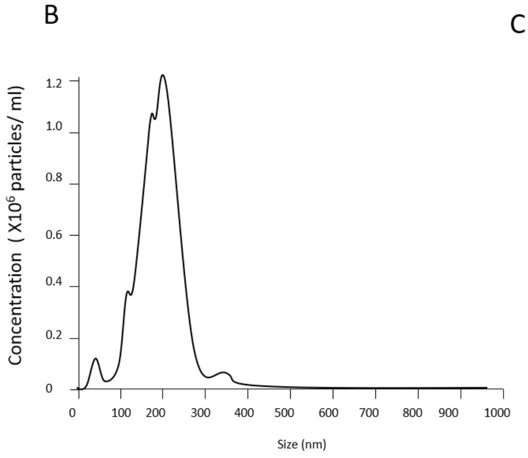

C

Exosome collecting time after transplant	Size of exosome (nm)
Day0	257.2 ± 87.9
Day3	225.9 ± 100.6
Day7	155.4 ± 66.9
Day10	232.7 ± 110.7
Day14	197.2 ± 59.4

Identification of exosomes isolated from serum using membrane markers and size distributions *via* a nanoparticle tracking analysis (NTA) system.

(A) Blotting images detected with anti-CD9, anti-CD63, and anti-CD81 antibodies of two samples (1 and 2) of exosomes isolated from the serum of mice on day 0 after transplantation of B16/BL6 cells; (B) representative size distribution of exosomes isolated from serum at day 0 after transplanting B16/BL6 cells using the NTA system; (C) particle sizes of each exosome from different serum collection time points after transplanting B16/BL6 cells by the NTA system. Data represent the mean ± SD of three independent experiments.

Supplementary Figure S2

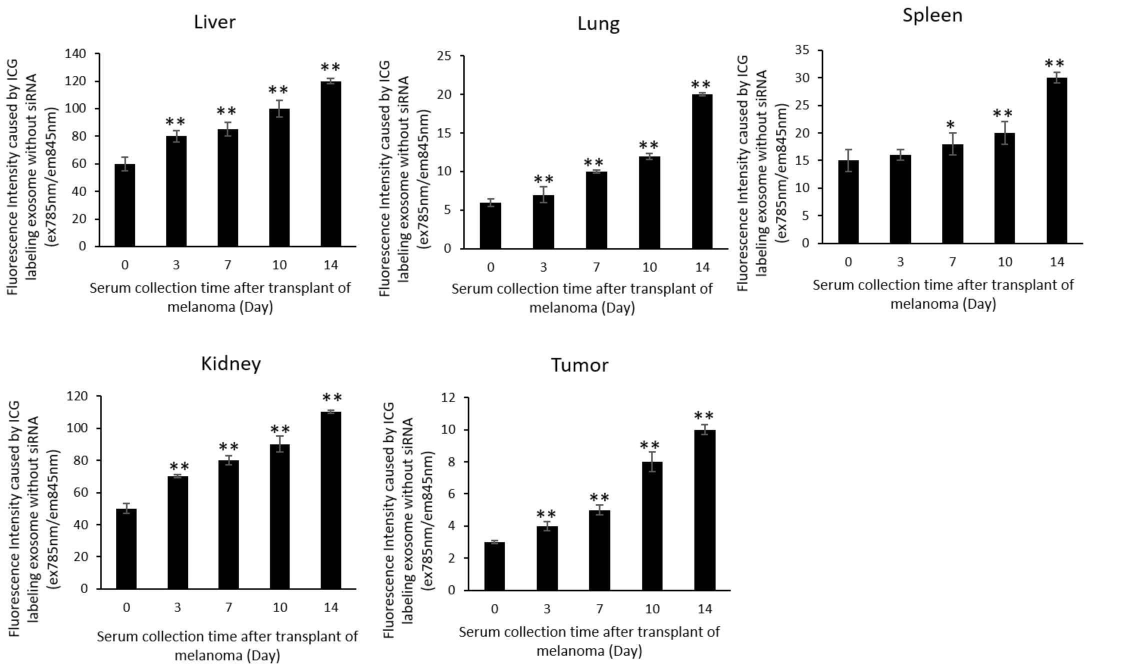

Distribution of ICG-labeled exosomes at 24 h after injection using samples from days 0 to 14 after subcutaneously transplanting B16/BL6 cells into the hind limb.

The fluorescence intensities of ICG-labeled exosomes from mice with tumors for the indicated number of days in five organs (liver, lung, spleen, kidney and tumor [hindlimb]) 24 h after injection. Data represent the mean ± SD of three independent experiments; ***p*<0.01 versus the day-0 exosome group (Bonferroni test/ANOVA).
